# Molecular approaches to the analysis of deformed wing virus replication and pathogenesis in the honey bee, *Apis mellifera*

**DOI:** 10.1186/1743-422X-6-221

**Published:** 2009-12-11

**Authors:** Humberto F Boncristiani, Gennaro Di Prisco, Jeffery S Pettis, Michele Hamilton, Yan Ping Chen

**Affiliations:** 1 USDA-ARS Bee Research Laboratory, Beltsville, MD 20705, USA; 2 Dipartimento di Entomologia e Zoologia Agraria "Filippo Silvestri" - Via Università n. °100, 80055 Portici, Napoli, Italy

## Abstract

**Background:**

For years, the understanding of the pathogenetic mechanisms that underlie honey bee viral diseases has been severely hindered because of the lack of a cell culture system for virus propagation. As a result, it is very imperative to develop new methods that would permit the *in vitro *pathogenesis study of honey bee viruses. The identification of virus replication is an important step towards the understanding of the pathogenesis process of viruses in their respective hosts. In the present study, we developed a strand-specific RT-PCR-based method for analysis of Deformed Wing Virus (DWV) replication in honey bees and in honey bee parasitic mites, *Varroa Destructor*.

**Results:**

The results shows that the method developed in our study allows reliable identification of the virus replication and solves the problem of falsely-primed cDNA amplifications that commonly exists in the current system. Using TaqMan real-time quantitative RT-PCR incorporated with biotinylated primers and magnetic beads purification step, we characterized the replication and tissue tropism of DWV infection in honey bees. We provide evidence for DWV replication in the tissues of wings, head, thorax, legs, hemolymph, and gut of honey bees and also in Varroa mites.

**Conclusion:**

The strategy reported in the present study forms a model system for studying bee virus replication, pathogenesis and immunity. This study should be a significant contribution to the goal of achieving a better understanding of virus pathogenesis in honey bees and to the design of appropriate control measures for bee populations at risk to virus infections.

## Background

The viruses pose a serious threat to the health and well-being of the honey bee, *Apis mellifera*, the most economically valuable pollinator of agricultural and horticultural crops worldwide. In the U.S. alone, the honey bee has an annual market value exceeding 14.6 billion dollars producing honey and other hive products [1]. So far, honey bees have been reported to be attacked by at least 18 viruses, most of which are single-strand positive sense RNA viruses [2,3]. Recently, honey bees have drawn significant attention to the scientific community and beekeeping industry due to the serious disease called Colony Collapse Disorder (CCD), a malady that has killed billions of bees since 2006 across the U.S. and around the world [4-6]. A study using a metagenomic approach found that Israeli Acute Paralysis Virus (IAPV), a species that was originally identified in honey bees in Israel showed that IAPV was detected in 25 of 30 (83%) CCD-affected honey bee colonies but in only one of 21 healthy colonies (Cox-Foster et al., 2007). The observed tight correclation between the IAPV and CCD affected colonies in the U.S. has raised serious concerns about risks of virus infections in honey bees. Although significant progress has been made in honey bee virus research in the last few decades (Reviewed in Chen and Siede, 2007 [7], investigation into virus replication and pathogenicity has been severely hindered because of the lack of a cell culture system for virus propagation. Therefore, observations of virus cytopathic effect (CPE) in cultured cells, a standard method used for unraveling the mechanisms of viral replication and the specific host responses to viral infections, are not possible. As a result, it is to develop new methods that would permit the study of virus replication *in vitro*.

Recent advances in molecular technology have greatly expanded our ability to detect and elucidate the molecular events associated with virus infections and pathogenesis. With the current molecular technology, complete genomes of several honey bee viruses have been sequenced and analyzed [8-14]. Using RT-PCR based assays, the virus infections in honey bees can be detected and quantified in a rapid and accurate manner [15,16]. As with all single-strand positive sense RNA viruses, replication of honey bee viruses proceeds via the production of a negative-strand intermediate and its presence is indicative of active viral replication. Therefore, the detection of negative-strand RNA of viruses offers an excellent alternative for studying virus replication and pathogenesis in naturally infected hosts [17]. Strand-specific RT-PCR was first developed for detection of negative-strand RNAs of viruses. However, the method has been reported to cause falsely-primed amplification due to the self priming of positive-strand RNA during reverse transcription or random priming by present contaminating cellular nucleic acids as tRNA, challenging the accuracy previous methods [18,19]. To overcome such occurrences, more effective techniques including Tagged RT-PCR, rTth RT-PCR and chemically blocking the free 3' ends of the RNA, have been developed to reduce nonspecific priming events [19-23]. In order to further improve the assay specificity, it was developed a new sensitive assay incorporating TaqMan quantitative RT-PCR with biotinylated primers and magnetic beads purification for detection of negative-strand viral RNAs. Furthermore, using the method developed, we analyzed replication and tissue tropism of Deformed Wing Virus (DWV), a highly prevalent honey bee virus that causes wing deformity and mortality in honey bees worldwide, in both bees with wing deformity (symptomatic infection) and bees with normal wings (asymptomatic infection). The replication of DWV in honey bee parasitic mites (*Varroa destructor*), a potential vector of DWV, was also investigated.

## Results

**Figure 1:**
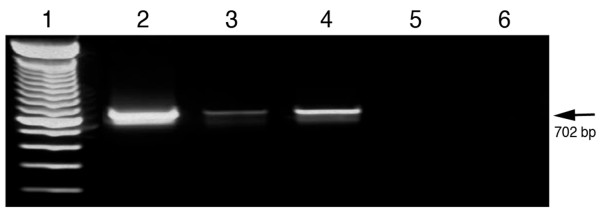
**Conventional RT-PCR for strand-specific detection of DWV RNA**. Total RNAs extracted from DWV-infected bees. Both negative and positive-strands of DWV RNA were specifically amplified by conventional RT-PCR using DWV-specific forward (line 2) and reverse primer (line 3), respectively, for initial reverse transcription. RT-PCR amplification, was also conducted without inclusion of any primers for reverse transcription (line 4). Negative controls containing no template (line 5) and no reverse transcriptase (line 6) yielded no products. The 100-bp ladder was loaded into lane 1. The arrow on the right indicates the expected 702 bp RT-PCR products.

The strand specificity of conventional RT-PCR was evaluated. As shown in Figure 1, both negative and positive-strands of DWV RNA templates were detected from bees with deformed wings using forward and reverse primers, respectively, for initial reverse transcription followed by amplification of the cDNA by PCR. The band intensity of negative-strand DWV fragments was significantly stronger than that of positive-strand DWV fragments. However, the DWV specific fragments were also amplified by RT-PCR without any primers for reverse transcription. Negative signals were obtained for negative control reactions without template or reverse transcriptase, confirming that RT-PCRs were not contaminated and were from the RNA templates.

**Figure 2:**
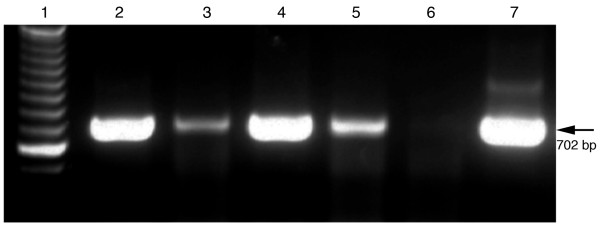
**Tagged RT-PCR for strand-specific detection of DWV RNA**. Total RNAs extracted from bees with deformed wings. The negative and positive-strand cDNAs that were generated by tag-forward primers or tag-reverse primers in reverse transcription, respectively, were consistently amplified by PCR using a pair of tag-primer and reverse primer (lane 2), a single reverse primer (lane 3), a pair of tag-primer and forward primer (lane 4), or a single forward primer (lane 5). Water was used as a negative control (lane 6) and a plasmid with DWV fragment was used as a positive control (lane 7). A 100-bp ladder was loaded into lane 1. The arrow on the right indicates the expected 702 bp RT-PCR products.

In this study, Tagged RT-PCR was evaluated for its specificity for amplification of both positive and negative-strand RNA from bees with deformed wings using four combinations of primers. Tagged RT-PCR assay was based on the generation of cDNAs by the primer containing a tag and further amplification of cDNA by a tag-primer and a primer complementary to the synthesized cDNA. The result showed that cDNAs generated by either tag-forward or tag-reverse primers were consistently amplified by subsequent PCR amplification, regardless of whether a pair of primers or only a single primer was used for PCR amplification. As shown in Figure 2, when a tag-forward primer was used to reverse transcribe the negative-strand of DWV RNAs, the synthesized cDNA could be amplified by PCR not only with the primer pair, tag-primer and reverse primer, but also with a single reverse primer. Meanwhile, when tag-reverse primer was used to reverse transcribe the positive-strand of DWV RNAs, the synthesized cDNA could be amplified by PCR with both primer pairs, tag-primer and forward primer, and with a single forward primer. No amplification was detected in the negative control (no template).

**Figure 3:**
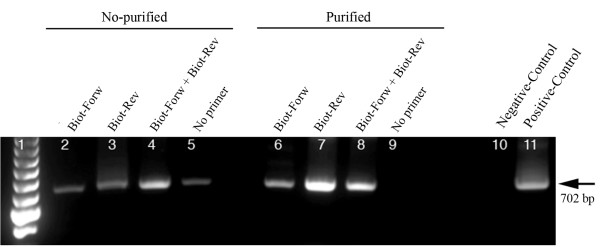
**RT-PCR incorporated with biotinylated primer and purification of magnetic beads for strand-specific detection of DWV RNA**. Total RNAs extracted from bees with deformed wings. The negative and positive-strand cDNAs that were generated by biotinylated forward (Lane 2 and 6) and reverse primers (Lane 3 and 7) in reverse transcription, respectively. The cDNA was generated by one step RT-PCR with both biotinylated forward and reverse primers (Lane 4 and 8). Reverse transcription was conducted without the addition of primer (Lane 5 and 9). The biotinylated cDNAs were either amplified directly by PCR (Lane 2-5) or subjected to magnetic bead purification before PCR amplification (Lane 6-9). Negative control without template (Lane 10) and positive control with recombinant DWV plasmid DNA (Lane 11) were included in the reaction. A 100-bp ladder was loaded into lane 1. The arrow on the right indicates the expected 702 bp RT-PCR products.

In order to achieve highly strand-specific detection of RNA for DWV, strand-specific RT-PCR was conducted using a biotinylated primer for cDNA generation and magnetic separation to purify synthesized cDNA prior to PCR amplification. As shown in Figure 3, without the magnetic separation step, cDNA generated using biotinylated forward, reverse or lack of primers for reverse transcription were all amplified by subsequent PCR, just like with conventional RT-PCR. However, the purification of biotinylated cDNA using streptavidin-coated magnetic beads excluded the non-specific amplification cDNAs that were spontaneously formed without the addition of primers for reverse transcription, which occurred in both conventional RT-PCR and Tagged RT-PCR.

**Figure 4:**
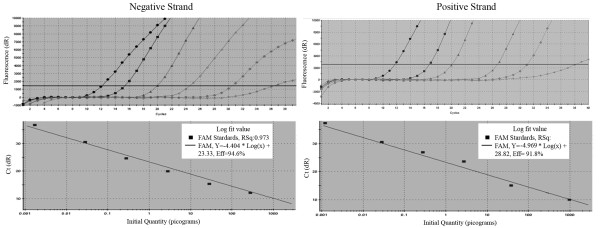
**Amplification plot and standard curve of TaqMan quantitative RT-PCR (qRT-PCR) incorporated with biotinylated primer and magnetic beads purification**. *In vitro *transcribed DWV RNA was serially diluted and subjected to RT-PCR to assess the sensitivity of the assay. A) Amplification plots were generated by using seven serial dilutions of the RNA, ranging from 10^3 ^pg to 1 fg per reaction as the template for the qRT-PCR assays. The amplification plot shows the fluorescence (dR) plotted against the cycle number for the standard dilution series of DWV. B) Standard curves were generated by plotting the observed C_T _value (Y axis) against the initial quantities of 10-fold serial diluted RNA. C_T _values are the average of three repetitions.

Further, the strand-specific TaqMan real-time qRT-PCR incorporated with biotinylated primer and magnetic separation was carried out for quantification of DWV replication in host tissues and parasitic mites of honey bees. To ensure an accurate quantification as well as the highest sensitivity of the assay, a standard curve was first established by plotting seven 10-fold dilutions of DWV-specific RNA *in vitro *transcribed from the pCR2.1 TA cloning vector against corresponding C_T _value. As shown in Figure 4A and 4B, a linear progression of the RT-PCR amplification was observed between the amount of input RNA ranging from 10^3 ^pg to 1 fg and the corresponding C_T _values within the concentration range. The detection limit of positive and negative-strand DWV RNAs were the same. The lowest limit of detection with qRT-PCR for DWV was 1 fg per reaction for both positive and negative-strand RNA. The RNA concentration below 1 fg could not be amplified in the reaction.

**Figure 5:**
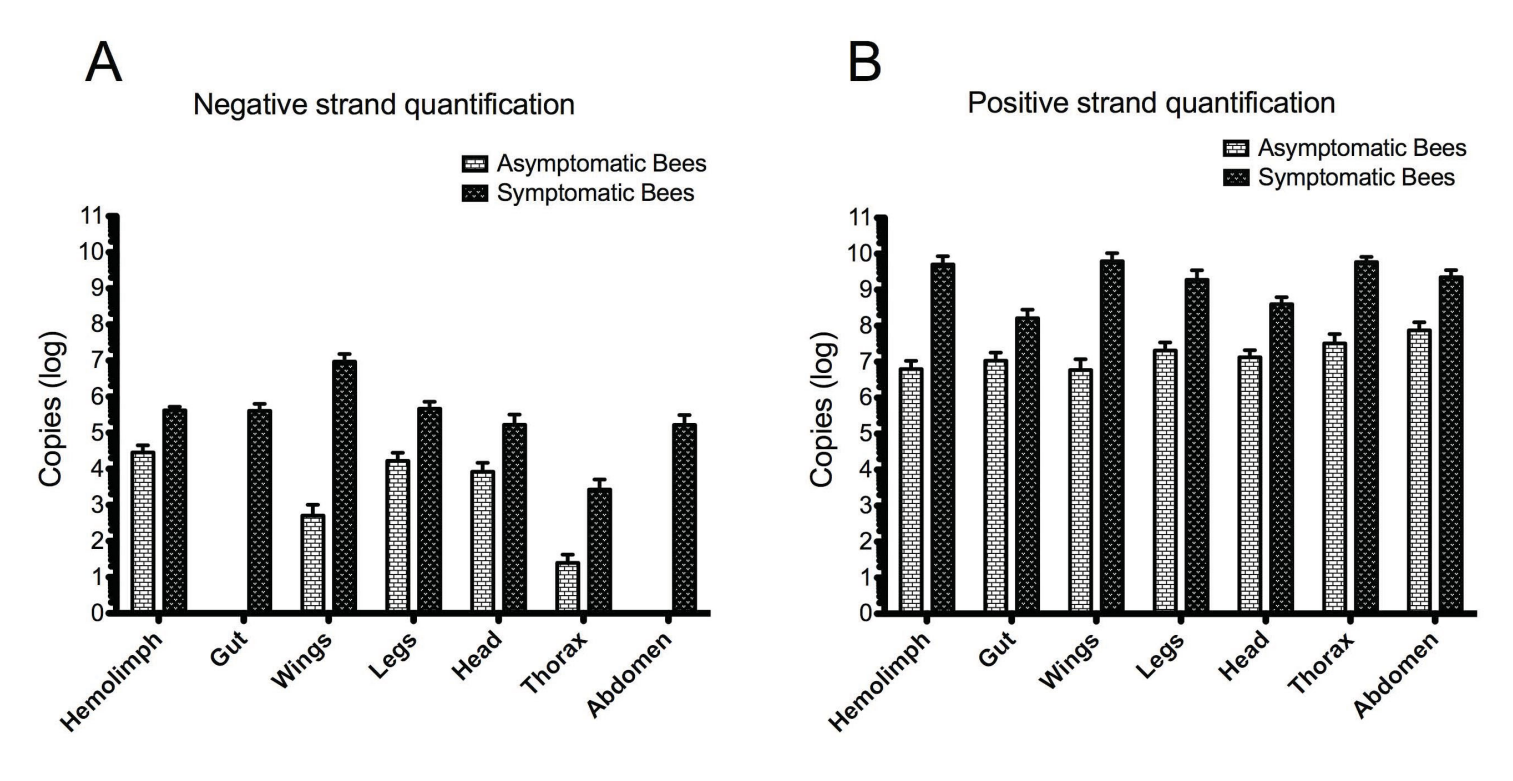
**TaqMan Real-Time qRT-PCR for quantification of negative and positive-strand DWV RNA in tissues of symptomatic and asymptomatic bees**. The hemolymph, gut, wings, legs, head, thorax and abdomen were individually dissected out from symptomatic and asymptomatic bees and subjected to TaqMan real-time qRT-PCR coupled with biotinylated primer and magnetic beads purification. The virus titer of negative-strand (A) and positive-strand (B) DWV of each sample was quantified with the standard curve and expressed as copy numbers (log).

The absolute quantification of positive and negative-strand DWV RNA in tissues of bees with symptomatic or asymptomatic infection and individual Varroa mites was carried out using a developed strand-specific assay. In bees with deformed wings, negative-strand DWV RNA was detected in all tissues examined. The concentration of negative-strand DWV RNA varied significantly among different tissues of the deformed bees (p < 0.001) and descended down in order of wings, hemolymph, legs, gut, head, abdomen, and thorax (Figure 5A). Meanwhile, except for the gut and abdomen, negative-strand DWV RNA was also detected in tissues of bees with normal wings. However, the average titer of negative-strand viral RNA in bees with wing deformity was 14.6 times higher than that in bees with normal wings in the hemolymph (p value < 0.0001), 1.8 × 10 ^4 ^times higher in the wings (p < 0.0001), 27.8 times higher in the legs (p < 0.0001), 19.8 times higher in the head (p = 0.0015), 107.1 times higher in the thorax (p = 0.0008) (Figure 5A).

Positive-strand DWV RNA showed predominant presence and the average positive-strand DWV RNA being 3 × 10^3 ^times more abundant than negative-strand DWV RNA in the virus-infected bees (p = 0.007). Positive-strand DWV RNA was detected in all tissues of bees with symptomatic or asymptomatic infections even though the average concentration of positive-strand viral RNA in tissues of bees with wing deformity was significantly higher than tissues of bees with normal wings; 8 × 10^2 ^times more in the hemolymph, 10^3 ^times more in the wings, 90 times more in the legs, 29.8 times more in the head, 1.8 × 10^3 ^times more in the thorax, 15.2 times more in the gut (p < 0.0001) and 29.8 times more in the abdomen (p < 0.0001) (Figure 5B).

Using the same methodology, negative-strand RNA of DWV was detected in 81% (17/21) while the positive-strand RNA of DWV was found in 95.2% (20/21) of the Varroa mites tested. Quantification of positive and negative-strand in the Varroa mites showed no significant difference (p = 0.07) between the titers of the negative-strand and positive-strand RNAs as seen in the honey bees (p < 0.05).

## Discussion

Replication is a key step in successful virus infections. The replication strategies of positive-strand RNA viruses share remarkable similarities: all replicate and express their genomes through negative-strand RNA intermediates that are used as templates for the production of positive-strand progeny RNAs packaged in new virion particles [24]. Therefore, the presence of negative-strand RNA intermediates should serve as a reliable marker for active virus replication in infected hosts.

In an attempt to develop an *in vitro *RNA replication assay for honey bee viruses, we first evaluated the existing methods, including conventional RT-PCR and Tagged RT-PCR for specificity of strand-specific detection of DWV RNA. The results showed that DWV RNA could be amplified by conventional RT-PCR without any primer for reverse transcription. The reason for nonspecific cDNA synthesis by conventional RT-PCR could be attributed to different events, including false-priming by antigenomic viral RNA or cellular RNAs, as well as self primering due to the secondary structure at the 5'UTR of viral RNA during reverse transcription, as earlier reports suggested [19,25-27]. Tagged RT-PCR was developed to resolve the problem of PCR amplification of falsely-primed cDNA associated with conventional RT-PCR [28,29]. However, the finding that cDNAs generated by tag-forward or tag-reverse primers were amplified by subsequent PCR with a single reverse primer or forward primer, respectively, suggested that the residue of tagged-primer from reverse transcription possibly served in subsequent PCR and led to amplification of non-strand-specific products, making necessary a purification step to assure total elimination of remaining primer from RT products or primer concentration reduction in RT reaction [28]. The evidence that conventional RT-PCR and Tagged RT-PCR without purification, failed to discriminate between the two strands of viral RNA in this study, suggests that caution should be taken with regards to the strand specificity of such methods.

In order to circumvent the problems of false priming and contamination of residue primers from RT, it is worthwhile to develop improved procedures for analysis of virus replication. We report here the development of a novel strand-specific RT-PCR coupled with a biotinylated primer for reverse transcription and purification of biotinylated cDNA with magnetic beads. Using biotinylated oligonucleotide primers during the reverse transcription can lead to subsequent synthesis of biotinylated cDNAs, which have a high binding affinity to the streptavidin-coated magnetic beads. The purification step of streptavidin magnetic beads-cDNA complex, ensures the capture of only biotinylated cDNAs. The disappearance of a positive signal for non-strand-specific amplification of magnetic-bead purified biotinylated cDNA in our study suggests that the assay developed is a significant modification of conventional or Tagged RT-PCR. The purification of biotinylated cDNA with magnetic beads is a key step in assuring the strand-specific detection and elimination of the residues of RNA, non-incorporated RT primers as well as enzymes and salts that would interfere with subsequent PCR amplification.

While quantitative detection of DWV infection and tissue tropism of DWV in the host were previously reported [30,31], there is lack of information on quantification of virus replicative intermediates and differentiation of positive and negative-strand DWV RNA in the different tissues of infected bees, which could provide important insight into the complexity of virus replication strategies leading to disease pathogenesis. To gain a better understanding of active sites of DWV replication in infected bees, we applied the method developed in our study to localize and quantify the positive and negative-strand DWV RNA in different host tissues. Our results showed that replication of DWV was spread throughout the body of bees with symptomatic infection. The detection of replicative intermediates, negative-strand DWV RNA, in the hemolymph, gut, wings, legs, head, thorax, and abdomen indicated that active replication occurred in these sites.

The observation that different tissues showed distinct kinetics of DWV replication, together with the fact that differences in the titers of positive-strand DWV RNAs were not significant among tissues examined, suggested that DWV had a tropism to certain host tissues in the replication. Among seven tissues examined, the most abundant amount of negative-strand DWV RNA was detected in the wings, which suggests that the wings are likely the predominant tissue site of DWV replication. Our earlier studies [15,31] demonstrated that colony foods could act as a vehicle for transmission of DWV and suggested that the lining of the digestive tract was likely the primary site of the DWV infection via food-borne transmission, with the virus spreading to secondary sites from the digestive tract. The presence of positive-strands, likely from food, and the absence of replication (negative-strands) of DWV RNA only in the gut and abdomen of asymptomatic bees, indicated that these sites are critical for the DWV pathogenesis course, signifying the necessity of an unknown event at these sites, such as a co-infection or a differential genetic background, to generate the permissive environment for a massive replication observed in symptomatic bees, likely responsible for the symptoms observed.

Compared to the titer of positive-strand DWV RNA, the relatively low amount of negative-strand DWV RNA implies that a regulatory mechanism may exist to facilitate the viral replication. These findings would stimulate further investigation to unveil the regulatory mechanisms that honey bees use to control the pattern of replication. Both positive and negative-strand RNAs of DWV were also detected in Varroa mites (data not shown) using the magnetic beads methodology. This finding is in agreement with preliminary works [22,23] and supports the conclusion that the Varroa mite may be a biological vector of DWV.

In sum, strand-specific detection and quantification of DWV RNA were achieved using qRT-PCR incorporated with biotinylated primers and purification of biotinylated cDNA with magnetic beads. The elucidation of DWV replication profiles in honey bees would have broad implications for future development of therapeutic strategies for viral diseases. The assay developed in this study represents a useful tool to study not only replication of honey bee viruses but also other single-stranded RNA viruses.

## Conclusions

We conclude that qRT-PCR incorporated with biotinylated primers and purification of biotinylated cDNAs with magnetic beads is a strong approach to specific detection and quantification of a virus genome and anti-genome *in vivo *using the honey bee as a model. This permitted the specific identification of an important honey bee virus (DWV) replication sites in the honey bee body and it's quantification. The screening between symptomatic and asymptomatic bees using this technology had permitted the identification of the digestive tract and abdomen as a critical replication site in the course of symptomatic infection.

## Methods

### Sample Preparation

Individual adult worker bees, with and without deformed wings, and individual Varroa mites, were collected from honey bee colonies that were left untreated for *Varroa *mites and maintained in the USDA-ARS Bee Research Laboratory backyard apiary, Beltsville, MD. Bees intended for tissue dissection were kept alive inside of containers with the cap loosened to allow airflow before dissection. Otherwise, bees were immediately stored in -80°C freezer for subsequent RNA extraction.

### Tissue Dissection

Each live bee (N = 18) was fixed on the wax top of a dissecting dish with insect pins. Under a dissecting microscope, hemolymph was collected with a micropipette by making a small hole at the joint area between the wing and body with a needle to make it bleed. Following hemolymph collection, the gut was carefully pulled out with forceps. The remaining body including wings, legs, head, thorax and abdomen were cut apart with scissors and collected individually. To prevent possible contamination with hemolymph, all tissues were thoroughly rinsed once with 1× PBS and twice with nuclease-free water. All the tissue samples were subjected to RNA extraction.

### RNA Extraction

Collected tissues from bees and Varroa mites (n = 21) were individually homogenized in TRIzol Reagent, a solution of guanidine isothiocyanate and phenol, for RNA extraction (Invitrogen, Carlsbad, CA). After the addition of chloroform to remove proteins, lipids and DNA, the upper aqueous phase containing RNA was removed to a new microcentrifuge tube, precipitated with isopropanol, and the resulting pellet was dissolved in diethyl-pyrocarbonate (DEPC)-treated water with the addition of 1 μl of RNaseOUT a ribonuclease inhibitor (Invitrogen, Carlsbad, CA). The concentration of total RNA was determined by measuring the absorption at 260 nm and the purity of RNA was estimated by the absorbance ratio of 260 nm/280 nm using a spectrophotometer with a 50 μl ultramicrovolume cell holder (Ultrospec 3300 *pro*, Amersham Biosciences). RNA samples were stored at -80°C prior to molecular detection for viruses.

### Conventional RT-PCR

The Access RT-PCR system (Promega, Madison, WI) was used for RT-PCR following the manufacturer's instructions. Primers used in the study were a pair of DWV specific primers as reported before [30]. In order to demonstrate the existence of falsely primed cDNAs amplification in the current detection system, the reverse transcriptions were conducted in the presence of 1 μM of forward primer or reverse primer for negative-strand RNA and positive-strand RNA, respectively. Reverse transcription without the addition of either forward or reverse primer in the reaction mixture was performed as a control. The reaction mixture contained: 1 × AMV/Tfl reaction buffer, 0.2 mM each dNTP, 1 μM primer, 2 mM MgSO4, 0.1 unit AMV reverse transcriptase, 0.1 unit Tfl DNA polymerase and 500 ng total RNA with a total volume of 25 μl. The reaction was conducted using the PTC-100 DNA Engine (MJ Research, Waltham, MA). The reverse transcriptions were performed at 48°C for 45 minutes. After the reverse transcription and inactivation of reverse transcriptase at 95°C for 5 minutes, the thermal machine was paused and the remaining primers (the reverse primer in the reaction with a forward primer, the forward primer in the reaction with a reverse primer, and both forward and reverse primers in the reaction without any primers) were added during the reverse transcription. The cDNAs were then amplified by PCR in the following thermal cycling profile: 40 cycles at 95°C for 30 sec, 55°C for 1 min, and 68°C for 2 min; 68°C for 7 min. Negative controls (water and a reaction without reverse transcriptase) were included in each run of RT-PCR. Amplified products were analyzed through determination of the size of PCR products by electrophoresis through a 1% agarose gel containing 0.5 ug/ml ethidium bromide and then visualized by UV transillumination. To prevent any potential contamination, pre-PCR set up and post-PCR analysis steps were carried out in separate rooms.

### Tagged RT-PCR

Tagged RT-PCRs were performed under the same thermal cycling conditions as the conventional RT-PCR described above, except that the primers used in the study were modified by adding a 15-bp long sequence tag [22]. The sequence of the tag was neither homologous nor complementary to the sequence of DWV. The reverse transcriptions were conducted with the addition of 1 μM of a tag-forward primer or a tag-reverse primer for negative-strand RNA and positive-strand RNA, respectively. After the reverse transcription reactions and the inactivation of reverse transcriptase, the tag and DWV-reverse primers or only the DWV reverse primer was added to the PCR reaction with cDNA generated by a tag-forward primer. The tag and DWV-forward primers or only the DWV forward primer was added to the PCR reaction with cDNA generated by a tag-reverse primer.

### Strand Specific RT-PCR

In order to increase strand specificity, the regular primers or tagged-primers were replaced by biotinylated-primers for reverse transcription. The biotinylated-primers were synthesized by Invitrogen. After the reverse transcription reaction, cDNAs generated by either biotinylated forward or biotinylated reverse primers were magnetically purified by the Dynabeads kilobaseBINDER M-280 kit (Invitrogen, Carlsbad, CA) following the manufacturer's recommendations. Magnetic beads coated with a monolayer of streptavidin were added to the RT reaction mixture containing the biotinylated cDNAs. The reaction mixture was incubated at room temperature for 3 hours in a roller incubator to allow immobilization of the biotinylated cDNA onto the magnetic beads. The Dynabeads/DNA-complex were washed twice in washing solution and once in distilled water and resuspended in PBS buffer. After releasing immobilized Biotinylated cDNAs from magnetic beads, PCR amplification was conducted for each sample under the same conditions as described above. The cDNAs that were generated by biotinylated primers and directly subjected to PCR amplification without magnetic bead purification were used as a control.

### Strand-Specific real-time TaqMan quantitative RT-PCR (qRT-PCR)

TaqMan real-time quantitative RT-PCR (qRT-PCR) incorporated with biotinylated-primers and magnetic bead purification was performed for quantification of negative and positive-strands of DWV using the Stratagene Mx3000P spectrofluorometric thermal cycler operated by MxPro qPCR software. The virus levels were quantified based on the value of the cycle threshold (Ct), which represents the number of cycles needed to generate a fluorescent signal above a predefined threshold and is inversely proportional to the concentration of the initial target that has been amplified. The house keeping gene, β-actin, was employed as an endogenous control for normalization of the quantification. The sequence information of primers and probes for both DWV and β-actin are the same as described before [30]. The amplification reaction mixture and conditions were the same as the strand-specific RT-PCR, mentioned above, except that a 0.2 μM TaqMan probe was incorporated in the PCR amplification. The measurement of the strand-specific virus titer was conducted in bees with deformed wings and with apparently normal wings.

### Standard Curve Establishment

Purified DWV specific fragments were incorporated into a pCR2.1 TA cloning vector (Invitrogen, Carlsbad, CA) following the manufacturer's protocol. Recombinant plasmid DNA, containing DWV fragment in both directions, was purified using the Plasmid Mini Prep Kit (BIO-RAD, Hercules, CA) and used as a template for *in vitro *transcription using the Megascript T7 kit (Applied Biosystems/Ambion, Austin, TX) in order to generate positive and negative DWV-specific ssRNAs and to construct a new standard curve for sensitive analysis and absolute virus quantification. Seven 10-fold serial dilutions of positive or negative (10^3 ^pg, 10^2 ^pg, 10^1 ^pg, 10^0 ^pg, 10^-1 ^pg, 10^-2 ^pg, and 10^-3 ^pg) in vitro transcribed RNA were subjected to reverse transcription with biotinylated primers followed by magnetic bead purification and qPCR amplification. Each sample was run in triplicate for statistical purposes. The standard curve was established by plotting the initial quantities of the 10-fold serial diluted RNA against the corresponding threshold value (C_T_).

### Sequencing

The specificity of the RT-PCR assay was confirmed by sequencing analysis. RT-PCR bands were excised from a low-melting-temperature agarose gel (Invitrogen, Carlsbad, CA) and purified using the Wizard PCR Prep DNA purification system (Promega, Madison, WI). The nucleotide sequences of the RT-PCR fragments were determined from both forward and reverse directions to confirm the specificity of the DWV amplification. The sequence data of each virus fragment were analyzed using the BLAST server at the National Center for Biotechnology Information, NIH.

### Statistical Analysis

The Fisher's Least Significant Difference (LSD) Comparison of Means Test was used to analyze for significant differences of positive and negative-strand DWV titers among different tissues of the bees. The results are expressed as mean ± SD. Differences were considered statistically significant if p value < 0.05.

## Abbreviations

DWV: Deformed Wing Virus; RT-PCR: Retro transcription-Polymerase chain reaction; CCD; Colony Collapse Disorder; CPE: Cytopathic effect.

## Competing interests

The authors declare that they have no competing interests.

## Authors' contributions

HFB conceived the research, performed the experiments, and wrote the manuscript. YPC developed the conceptual aspects of the work and wrote and edited the manuscript. All authors participated in data collection and read and approved the final manuscript.

## Disclaimer

Mention of trade names or commercial products in this article is solely for the purpose of providing specific information and does not imply recommendation or endorsement by the U.S. Department of Agriculture.

## References

[B1] Morse RA Calderone M The value of honey bee pollination in the United States Bee Culture 2000 128 1 15

[B2] Allen M Ball B The incidence and world distribution of honey bee viruses Bee World 1996 77 141 162

[B3] Ellis JD Munn PA The worldwide health status of honey bees Bee World 2005 86 88 101

[B4] Cox-Foster DL Conlan S Holmes EC Palacios G Evans JD Moran NA Quan PL Briese T Hornig M Geiser DM A metagenomic survey of microbes in honey bee colony collapse disorder Science 2007 318 283 287 10.1126/science.1146498 17823314

[B5] Vanengelsdorp D Underwood R Caron D Hayes J An estimate of managed colony losses in the winter of 2006-2007: A report commissioned by the apiary inspectors of America American Bee Journal 2007 147 599 603

[B6] Vanengelsdorp D Evans JD Saegerman C Mullin C Haubruge E Nguyen BK Frazier M Frazier J Cox-Foster D Chen Y Colony Collapse Disorder: A Descriptive Study PLoS ONE 2009 4 e6481 10.1371/journal.pone.0006481 19649264 PMC2715894

[B7] Chen YP Siede R Honey bee viruses Adv Virus Res 2007 70 33 80 10.1016/S0065-3527(07)70002-7 17765703

[B8] Ghosh RC Ball BV Willcocks MM Carter MJ The nucleotide sequence of sacbrood virus of the honey bee: an insect picorna-like virus J Gen Virol 1999 80 Pt 6 1541 1549 10374974 10.1099/0022-1317-80-6-1541

[B9] Govan VA Leat N Allsopp M Davison S Analysis of the complete genome sequence of acute bee paralysis virus shows that it belongs to the novel group of insect-infecting RNA viruses Virology 2000 277 457 463 10.1006/viro.2000.0616 11080493

[B10] Leat N Ball B Govan V Davison S Analysis of the complete genome sequence of black queen-cell virus, a picorna-like virus of honey bees J Gen Virol 2000 81 2111 2119 10900051 10.1099/0022-1317-81-8-2111

[B11] Lanzi G de Miranda JR Boniotti MB Cameron CE Lavazza A Capucci L Camazine SM Rossi C Molecular and biological characterization of deformed wing virus of honeybees (Apis mellifera L.) J Virol 2006 80 4998 5009 10.1128/JVI.80.10.4998-5009.2006 16641291 PMC1472076

[B12] Maori E Lavi S Mozes-Koch R Gantman Y Peretz Y Edelbaum O Tanne E Sela I Isolation and characterization of Israeli acute paralysis virus, a dicistrovirus affecting honeybees in Israel: evidence for diversity due to intra- and inter-species recombination J Gen Virol 2007 88 3428 3438 10.1099/vir.0.83284-0 18024913

[B13] de Miranda JR Drebot M Tyler S Shen M Cameron CE Stoltz DB Camazine SM Complete nucleotide sequence of Kashmir bee virus and comparison with acute bee paralysis virus J Gen Virol 2004 85 2263 2270 10.1099/vir.0.79990-0 15269367

[B14] Olivier V Blanchard P Chaouch S Lallemand P Schurr F Celle O Dubois E Tordo N Thiery R Houlgatte R Ribiere M Molecular characterisation and phylogenetic analysis of Chronic bee paralysis virus, a honey bee virus Virus Res 2008 132 59 68 10.1016/j.virusres.2007.10.014 18079012

[B15] Chen Y Evans J Feldlaufer M Horizontal and vertical transmission of viruses in the honey bee, Apis mellifera J Invertebr Pathol 2006 92 152 159 10.1016/j.jip.2006.03.010 16793058

[B16] Chen Y Pettis JS Feldlaufer MF Detection of multiple viruses in queens of the honey bee Apis mellifera L J Invertebr Pathol 2005 90 118 121 10.1016/j.jip.2005.08.005 16214161

[B17] Lanford RE Sureau C Jacob JR White R Fuerst TR Demonstration of in vitro infection of chimpanzee hepatocytes with hepatitis C virus using strand-specific RT/PCR Virology 1994 202 606 614 10.1006/viro.1994.1381 8030225

[B18] Haddad F Qin AX Giger JM Guo H Baldwin KM Potential pitfalls in the accuracy of analysis of natural sense-antisense RNA pairs by reverse transcription-PCR BMC Biotechnol 2007 7 21 10.1186/1472-6750-7-21 17480233 PMC1876213

[B19] Boncristiani HF Rossi RD Criado MF Furtado FM Arruda E Magnetic purification of biotinylated cDNA removes false-priming and ensures strandspecificity of RT-PCR for enteroviral RNAs Journal of Virological Methods 2009 161 147 153 10.1016/j.jviromet.2009.06.006 19524621

[B20] Lanford RE Sureau C Jacob JR White R Fuerst TR Demonstration of in-Vitro Infection of Chimpanzee Hepatocytes with Hepatitis-C Virus Using Strand-Specific Rt/Pcr Virology 1994 202 606 614 10.1006/viro.1994.1381 8030225

[B21] Gunji T Kato N Hijikata M Hayashi K Saitoh S Shimotohno K Specific detection of positive and negative-stranded hepatitis C viral RNA using chemical RNA modification Arch Virol 1994 134 293 302 10.1007/BF01310568 7510473

[B22] Yue C Genersch E RT-PCR analysis of Deformed wing virus in honeybees (Apis mellifera) and mites (Varroa destructor) J Gen Virol 2005 86 3419 3424 10.1099/vir.0.81401-0 16298989

[B23] Gisder S Aumeier P Genersch E Deformed wing virus: replication and viral load in mites (Varroa destructor) J Gen Virol 2009 90 463 467 10.1099/vir.0.005579-0 19141457

[B24] Racaniello V Kinipe DMHP Picornaviridae: the viruses and their replication Fields Virology. Philadelphia 2001 01 4 685 722

[B25] McGuinness PH Bishop GA McCaughan GW Trowbridge R Gowans EJ False detection of negative-strand hepatitis C virus RNA Lancet 1994 343 551 552 10.1016/S0140-6736(94)91509-1 7509432

[B26] Mellor J Haydon G Blair C Livingstone W Simmonds P Low level or absent in vivo replication of hepatitis C virus and hepatitis G virus/GB virus C in peripheral blood mononuclear cells J Gen Virol 1998 79 Pt 4 705 714 9568964 10.1099/0022-1317-79-4-705

[B27] Peyrefitte CN Pastorino B Bessaud M Tolou HJ Couissinier-Paris P Evidence for in vitro falsely-primed cDNAs that prevent specific detection of virus negative-strand RNAs in dengue-infected cells: improvement by Tagged RT-PCR J Virol Methods 2003 113 19 28 10.1016/S0166-0934(03)00218-0 14500123

[B28] Bessaud M Autret A Jegouic S Balanant J Joffret ML Delpeyroux F Development of a Taqman RT-PCR assay for the detection and quantification of negatively stranded RNA of human enteroviruses: evidence for false-priming and improvement by Tagged RT-PCR J Virol Methods 2008 153 182 189 10.1016/j.jviromet.2008.07.010 18706930

[B29] Craggs JK Ball JK Thomson BJ Irving WL Grabowska AM Development of a strand-specific RT-PCR based assay to detect the replicative form of hepatitis C virus RNA J Virol Methods 2001 94 111 120 10.1016/S0166-0934(01)00281-6 11337045

[B30] Chen YP Higgins JA Feldlaufer MF Quantitative real-time reverse transcription-PCR analysis of deformed wing virus infection in the honeybee (Apis mellifera L.) Appl Environ Microbiol 2005 71 436 441 10.1128/AEM.71.1.436-441.2005 15640219 PMC544241

[B31] Chen YP Pettis JS Collins A Feldlaufer MF Prevalence and transmission of honeybee viruses Appl Environ Microbiol 2006 72 606 611 10.1128/AEM.72.1.606-611.2006 16391097 PMC1352288

